# Chromosome-Level Genome Assembly and Comparative Genomic Analysis of *Quercus oxyphylla*, an Evergreen Subalpine Oak Species Endemic to China

**DOI:** 10.3390/plants15081238

**Published:** 2026-04-17

**Authors:** Jing-Yu Yang, Ying Fu, Chun-Ming Chen, Jun-Shu Ma, Lin-Rui Liu, Jia Yang

**Affiliations:** Key Laboratory of Resource Biology and Biotechnology in Western China, Ministry of Education, College of Life Sciences, Northwest University, Xi’an 710069, China; 202421732@stumail.nwu.edu.cn (J.-Y.Y.); 202332773@stumail.nwu.edu.cn (Y.F.); 202322563@stumail.nwu.edu.cn (C.-M.C.); 202121665@stumail.nwu.edu.cn (J.-S.M.); 202433603@stumail.nwu.edu.cn (L.-R.L.)

**Keywords:** genome assembly, *Quercus oxyphylla*, oak, genome evolution, LTR-RT insertion, gene family

## Abstract

*Quercus oxyphylla* (E. H. Wilson) Hand.-Mazz. is a threatened evergreen subalpine tree species with fragmented habitats native to China. Here, we present a de novo chromosome-level genome assembly of this oak species by integrating PacBio long-read high-fidelity (HiFi) sequencing and Hi-C mapping technologies. The assembled genome size of *Q. oxyphylla* in this study is 824.15 megabases (Mb) in length with 12 putative chromosomes. Genome annotation of this oak species identified 514.09 Mb of repeat sequences, 53,730 protein-coding genes and 1048 non-coding RNA sequences. Genomic analyses of whole-genome duplication (WGD) and long terminal repeat retrotransposon (LTR-RT) insertion events in *Q. oxyphylla* revealed no species-specific WGD and recent accumulation of LTR-RTs in the genome within the last seven million years. A phylogenomic analysis with eight oak representatives confirmed the framework phylogeny of genus *Quercus* and indicated that *Q. oxyphylla* possibly split with the ancestor of *Cerris* oaks about 20.4 million years ago. We identified 2074 expanded and 903 contracted gene families across the genome assembly of *Q. oxyphylla*, while the significantly expanded gene families had notable disease resistance-related genes that were mainly enriched in plant–pathogen interaction pathways. The high-quality genome assembly of *Q. oxyphylla* generated in this study provides a valuable genome resource for the genetic conservation and management of *Q. oxyphylla*, and may facilitate our understanding of genome evolution and species adaptation of the oak lineage.

## 1. Introduction

The knowledge of genomic information in organisms provides critical insight into patterns of species evolution on the tree of life [1,2,3]. Because of the great advances in sequencing biotechnology, for example, the applications of long-read DNA sequencing technologies and chromosome conformation capture protocols in sequence generation and accurate genome assembly, numerous high-quality genomic resources such as transcriptomes and organelle and nuclear genomes have been published and made available during the past few decades [4,5,6]. The huge sizes of genetic datasets, especially at the level of genomics, can promote the understanding of the genetic basis of species adaptation and lineage diversification in model and non-model organisms, and provide important evidence for conservation strategies for biodiversity on earth [3,5,7,8,9].

The oak genus (genus *Quercus* L.) is in fact a potential model for the study of adaptive evolution and speciation in long-lived plant species [10,11]. This genus comprises one of the most rich species lineages in Fagaceae, mostly consisting of keystone tree species in forests across the Northern Hemisphere with wide-ranging geographical distributions and a strong capacity for ecological adaptation, which provides important resources for ecology and forestry [10,12,13]. About ten years ago, the first genome sequence of *Quercus robur* L., a representative of white oak species, was decoded with an estimated genome size of 1.5 gigabases (Gb) using a whole-genome shotgun approach [14]. Studies on subsequently released oak genomes with high qualities such as *Quercus suber* L. [15,16], *Quercus lobata* Nee [17,18], *Quercus rubra* L. [19], *Quercus mongolica* Fisch. [20], *Quercus dentata* Thunb. [21], *Quercus variabilis* Blume [22], *Quercus acutissima* Carruth. [23,24] and *Quercus glauca* (Thunb.) Oerst. [25] have described the classic structures and characteristics of the oak genome; for example, oaks are diploid species with 12 chromosomes (2n = 2x = 24) and relatively high heterozygosity in their genomes [13,26]. According to DNA C-values, the estimated genome sizes of oak species are variant with ranges covering about 560 to 1200 megabases (Mb) [27]. Moreover, a number of identified genes and gene families associated with possible functions in longevity and ecological resistance are key to understanding the patterns of species adaptation and lineage diversification in oak species [13,14,15,20,28]. The advance of oak genomics is proving its worth and revealing significance in biological and ecological studies of the genus *Quercus* [14,27,29].

*Quercus oxyphylla* (E. H. Wilson) Hand.-Mazz. is an evergreen subalpine tree species endemic to China with special tolerance to dry limestone habitats [30]. According to our field investigation, current populations of *Q. oxyphylla* are extremely scattered in southwestern, central and eastern montane regions in China with relatively small population sizes. Most populations of *Q. oxyphylla* are facing a risk of extinction due to drastic human activity and climate change in species habitats, and this species is considered near-threatened in the IUCN (International Union for Conservation of Nature) red list (www.iucnredlist.org) (accessed on 1 May 2020). The evergreen *Q. oxyphylla* is morphologically distinct in terms of leaf and acorn characteristics, while previous phylogenetic reconstructions of Chinese oak representatives have identified this species in *Quercus* section *Ilex*, a species-rich group of the Old World oak clade, probably originating from the eastern Himalayan region [30,31,32]. However, to date, few genetic studies have been performed on *Q. oxyphylla*, which contributes little to the understanding of species genetic basis and population genetic differentiation as well as the genetic structure of this endemic oak species. Additionally, among the available oak genome references, most are widespread oak species from the *Quercus* sections *Quercus* and *Cerris*, while genome resources of *Ilex* oaks in the Old World oak clade are rare.

In this study, sequencing data from the long-read high-fidelity (HiFi) and Hi-C libraries were generated to assemble a chromosome-level genome of *Q. oxyphylla*, and the genomic features of this oak species were depicted through genome annotation. We also performed a comparative genomic analysis on the genome assembly to explore the patterns of genomic evolution and potential mechanism of species adaptation in *Q. oxyphylla*. The high-quality de novo genome assembly of *Q. oxyphylla* presented in this study may provide critical implications for future genetic conservation and management and population genomics of this endemic oak species, and also provide a candidate reference genome for the ecologically and phylogenetically important *Ilex* oaks of the genus *Quercus*.

## 2. Results

### 2.1. De Novo Assembly and Quality Evaluation on the Genome

A total of 5.5 million circular consensus sequencing (CCS) reads with 88.81 Gb high-quality sequencing data and 351 million clean raw reads with 104.89 Gb sequencing data were obtained from long-read HiFi and Hi-C libraries for the genome assembly of *Q. oxyphylla*, respectively. The estimated N50 length of the CCS long reads was 16.59 kilobases (kb), while the Q30 value of the Hi-C data was 0.9470. Additionally, we yielded 5.80 Gb high-quality transcriptome sequences containing about 19 million reads with Q30 > 0.93 for genome annotation of *Q. oxyphylla* in this study (Table S1). Using a K-mer analysis on the long-read HiFi dataset, the genome size of *Q. oxyphylla* was estimated to be about 737.35 Mb with 1.94% heterozygosity in the genome (K = 21, Figure S1).

**Figure 1:**
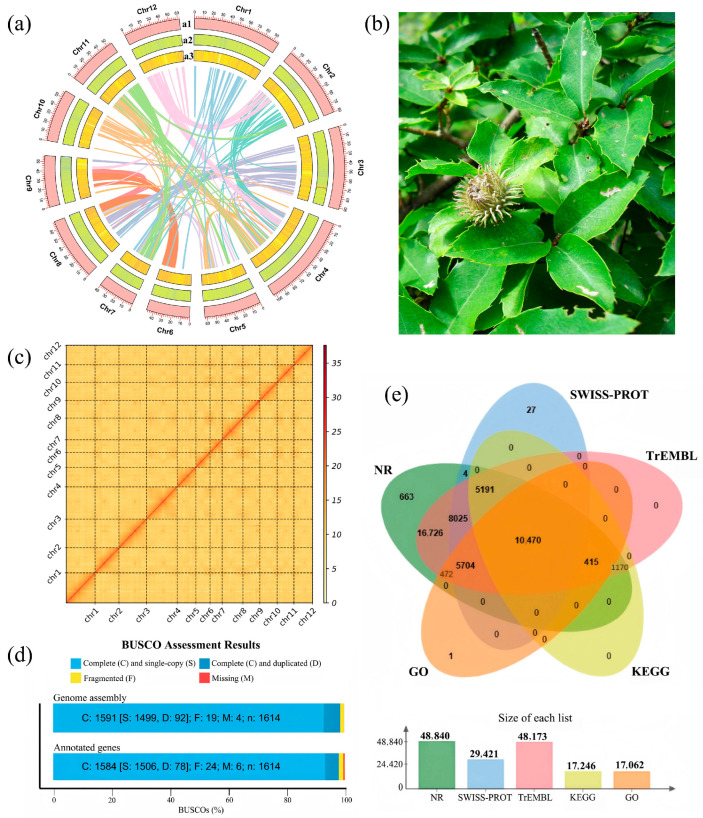
Assembly features of the *Q. oxyphylla* genome. (**a**) Circos view of the genomic information of *Q. oxyphylla* including assembled lengths of 12 putative chromosomes (a1), GC content (a2) and identified gene density (a3); links in the core indicate syntenic blocks between chromosomes. (**b**) Species image showing the characteristics of leaves and acorns of *Q. oxyphylla*. (**c**) Heatmap of chromosomal interaction in the genome assembly. (**d**) Results of BUSCO (benchmarking universal single-copy orthologs) assessment on the genome assembly and annotated genes of *Q. oxyphylla*. (**e**) Venn diagram of functional gene annotation on the genome assembly of *Q. oxyphylla* in five protein databases.

Based on the HiFi long reads and Hi-C sequencing data, we performed three rounds of assembly on the genome of *Q. oxyphylla*, which generated the assembled genome sizes of about 896 Mb (preliminary assembly), 855 Mb (redundancy removed) and 824 Mb (final assembly) for the studied oak species, respectively (Table 1). Briefly, we obtained a final chromosome-level assembly with a genome size of 824.15 Mb and 12 putative chromosomes for *Q. oxyphylla* (Figure 1a–c); the GC content of the final genome assembly was 35.74%, while the estimated scaffolds N50 and N90 were 69 Mb and 47 Mb, respectively (Table 1); the estimated lengths of the 12 assembled chromosomes ranged from 40 Mb (Chr7) to 102 Mb (Chr4), while the GC contents of the 12 chromosomes were similar, with values ranging from 35.46% (Chr1) to 36.37% (Chr9) (Table 2).

The heatmap of modified Hi-C results showed significant interaction and coherence of captured contigs and scaffolds within the 12 putative chromosomes of the genome assembly of *Q. oxyphylla* (Figure 1c). BUSCO (benchmarking universal single-copy orthologs) analyses on the genome assembly and annotated gene sets revealed that 98.57% and 98.14% of the genes were completely identified based on an embryophyte gene database (embryophyta_odb10), suggesting a high level of genome completeness in the final genome assembly in this study (Figure 1d). Furthermore, based on the annotated long terminal repeat (LTR) elements, the estimated LAI (LTR assembly index) value on the genome assembly was 18.39, while LAI scores of the 12 chromosomes ranged from 13.87 (Chr2) to 23.75 (Chr9), reflecting a candidate reference genome generated for *Q. oxyphylla* (Table 2).

### 2.2. Genome Annotation of Q. oxyphylla

A total of 1,766,678 repeat sequences were identified in the genome assembly of *Q. oxyphylla*, accounting for 62.38% (514.09 Mb) of the studied oak genome. Among the annotated repeats, the LTR retroelements were dominant (37.21%) with Ty1/*Copia* (12.53%) and *Gypsy*/DIRS1 (15.35%) representing the most abundant subtypes in the assembled genome. The DNA transposons only contributed 5.78% of the repeat sequences in the *Q. oxyphylla* genome (Table 3).

We identified 53,730 protein-coding genes with a mean of 3.98 exons per gene from the assembled genome of *Q. oxyphylla* based on an integration of three annotation strategies including ab initio, homology and transcriptome-based gene predictions. The average lengths of these annotated genes, coding DNA sequences (CDS) and exon and intron regions were 3548.05, 1026.24, 257.83 and 846.17 base pairs (bp), respectively (Table 4). Among the 12 chromosomes of the genome assembly, the largest number of genes was 7076, identified on chromosome 4 (Chr4), while chromosome 7 (Chr7) possessed the lowest number of genes (2937) in the *Q. oxyphylla* genome assembly (Table 2).

Further functional gene annotations were performed on the identified protein-coding sequences against the NR (Non-Redundant Protein Sequence Database), Swiss-Prot, TrEMBL, GO (Gene Ontology) and KEGG (Kyoto Encyclopedia of Genes and Genomes) databases, and a total of 50,210 functional genes (93.45% of the identified protein-coding genes) were acquired. Among the five protein databases, NR database got the highest proportion of identification (90.90%) with the functional annotation of genes in the genome assembly used in this study, while the GO and KEGG databases revealed relatively low proportions of annotation, with 31.76% and 32.10% functional genes assigned to the genome, respectively (Figure 1e and Table S2).

The identification of non-coding RNA sequences revealed 775 transfer RNAs (tRNAs) and 273 ribosomal RNAs (rRNAs) found in the genome assembly of *Q. oxyphylla*. The mean lengths of these two types of non-coding RNAs were 87.54 and 1390.30 bp, respectively.

### 2.3. Whole-Genome Duplication (WGD) and Long Terminal Repeat Retrotransposon (LTR-RT) Insertion Events in Q. oxyphylla

**Figure 2:**
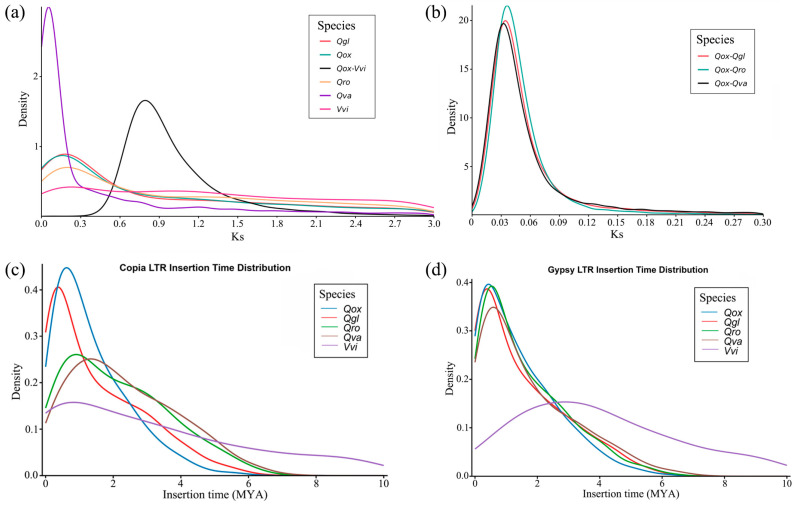
Genome evolution analysis of *Q. oxyphylla* with histogram distributions of (**a**,**b**) synonymous substitution rates (Ks) for whole-genome duplication and (**c**,**d**) insertion time estimations of two intact long terminal repeat (LTR) subtypes, *Copia* and *Gypsy*, in four related oak species including *Q. oxyphylla* (Qox), *Q. glauca* (Qgl), *Q. robur* (Qro), *Q. variabilis* (Qva) and grape (Vvi).

The WGD analysis using homologous gene pairs identified among four related oak species and grape (*Vitis vinifera* L.) (Table S3) revealed a single distributed peak of synonymous substitution rate (Ks) values around 0.1 to 0.2 in each species, suggesting a similar pattern of genomic evolution in *Q. oxyphylla* to other oaks as well as grape without a species-specific WGD event after the inferred divergence between oak species (*Q. oxyphylla*) and *V. vinifera* at a Ks value of 0.85 (Figure 2a). Further estimations of the distribution of Ks values between species pairs indicated almost simultaneous divergence times among *Q. oxyphylla* (Qox), *Q. glauca* (Qgl) and *Q. variabilis* (Qva) approximate to a Ks value of 0.04, slightly later than the inferred speciation event between *Q. oxyphylla* and *Q. robur* (Qro) at a Ks of 0.05; the inferred split between *Q. oxyphylla* (Qox) and *Q. variabilis* (Qva) was a little later than the estimated species divergence between *Q. oxyphylla* (Qox) and *Q. glauca* (Qgl) (Figure 2b).

The potential LTR insertion events in *Q. oxyphylla* were inferred with intact LTR-RT sequences identified in the genome assembly and compared with those of the three related oaks and grape (Table S4). The results of the LTR-RT insertion time distributions indicated a consistent evolutionary history of LTR accumulation in two major LTR elements (*Copia* and *Gypsy*) for the four studied oak species. Compared with grape, the insertion times of LTRs in the genome assembly of *Q. oxyphylla* as well as the other three related oaks were recent and probably accumulated seven million years ago (Mya) (Figure 2c,d).

### 2.4. Comparative Genomics on Phylogeny and Gene Families

**Figure 3:**
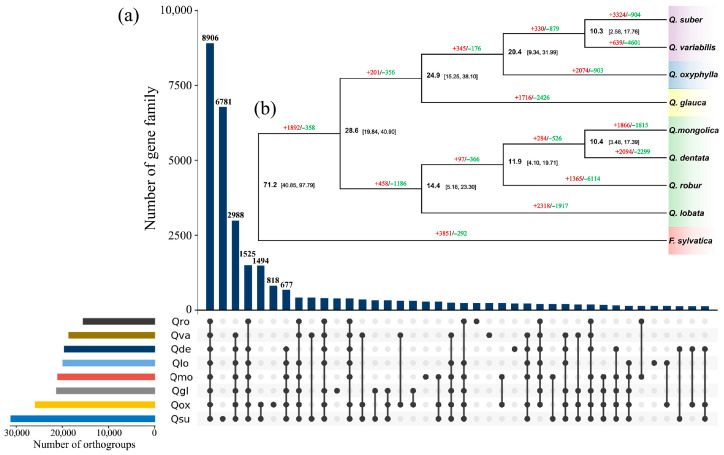
Comparative genomic analysis of *Q. oxyphylla*. (**a**) Upset diagram showing the results of identified gene families among eight oak species including *Q. oxyphylla* (Qox), *Q. robur* (Qro), *Q. variabilis* (Qva), *Q. dentata* (Qde), *Q. lobata* (Qlo), *Q. mongolica* (Qmo), *Q. glauca* (Qgl) and *Q. suber* (Qsu) used for phylogenetic analysis; numbers of identified gene families more than 500 are shown. (**b**) Phylogenetic tree with estimated divergence times on the node and identified gene families underlying expansion (numbers in red) and contraction (numbers in green) on the branch for eight oak species and the outgroup *F. sylvatica* L. All nodes on the phylogeny were strongly supported by bootstrap values of 100%.

A total of 193,288 orthogroups including 365,868 genes and 40,454 shared gene families were identified among eight oak species and the outgroup *Fagus sylvatica* L. used for phylogenetic analysis. For *Q. oxyphylla*, 25,849 orthogroups with 50,601 genes were classified, while 714 orthologous gene clusters were species-specific to the genome assembly (Table S5). The eight related oak species of the genus *Quercus* shared 8906 gene families with the identified orthogroups, while 818 gene families were found specific to *Q. oxyphylla* (Figure 3a).

Phylogenetic analysis was performed with single-copy genes from the identified gene families among the nine studied species. In the well-documented phylogenetic tree with 100% bootstrap supports on all nodes, *Q. oxyphylla* was assigned an independent species member in the Old World oak clade, clustering with *Q. glauca* from *Quercus* section *Cyclobalanopsis* and two oak siblings (*Q. suber* and *Q. variabilis*) from *Quercus* section *Cerris*. The mean time of divergence between *Q. oxyphylla* and *Cerris* oaks was estimated to be 20.4 Ma (95% highest posterior density: 9.34–31.99 Ma) (Figure 3b).

Further evaluations of shared gene families in the phylogeny indicated 2074 and 903 gene families underlying potential expansion and contraction in the assembled genome of *Q. oxyphylla*, respectively (Figure 3b). Among these identified gene families, 1111 expanded and 228 contracted gene families were found to be significant (*p*-value < 0.05), which included 5592 and 1741 candidate genes (Tables S6 and S7), respectively. The RNase H enzyme-encoding genes (268) and plant disease resistance-related genes (R-genes) including the NB-LRR (nucleotide-binding site and leucine-rich repeat) genes (174), LRR receptor-like genes (165), RLK (receptor-like protein kinase) genes (82) and other uncharacterized disease resistance genes (80) were notably identified in the significantly expanded gene families (Table S6).

**Figure 4:**
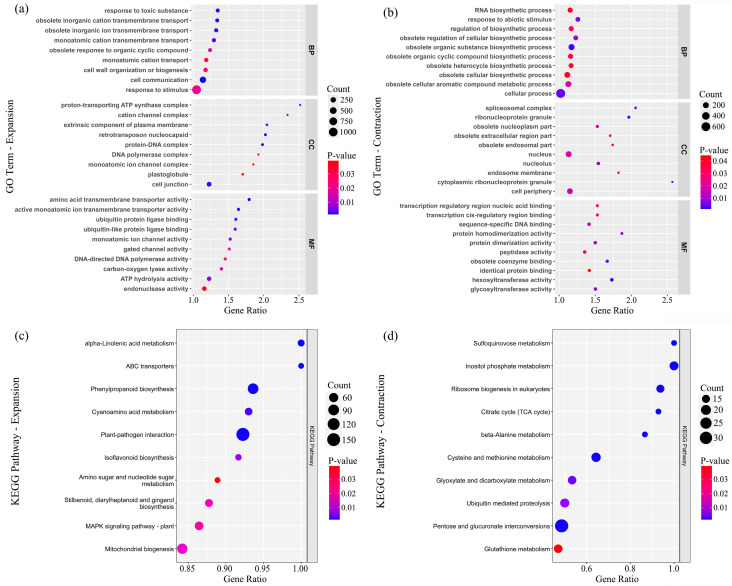
Top 10 results of (**a**,**b**) GO (Gene Ontology) and (**c**,**d**) KEGG (Kyoto Encyclopedia of Genes and Genomes) pathway enrichment analyses on significantly expanded and contracted gene families identified in the genome assembly of *Q. oxyphylla*.

Using GO enrichment analysis, the identified expanded and contracted gene families revealed significant enrichment in 248 and 194 GO terms of three functional categories including biological process (BP), molecular function (MF) and cellular component (CC) (adjusted *p*-value < 0.05) (Figure 4a,b and Tables S8 and S9). For example, functions associated with response to toxic substances and stimuli as well as transmembrane transport of obsolete inorganics in biological processes were assigned to the significantly expanded gene families; meanwhile, for the contracted gene families, a GO term related to response to abiotic stimuli in biological processes was notably enriched. For the KEGG pathway analysis, the expanded gene families had 21 significantly enriched pathways (adjusted *p*-value < 0.05) including the plant–pathogen interaction, alpha-Linolenic acid metabolism and ABC transporter pathways (Figure 4c and Table S10); the contracted gene families revealed 24 significant pathways (adjusted *p*-value < 0.05), most of which were related to organic substance metabolism (Figure 4d and Table S11).

## 3. Discussion

The oak genus is considered an outstanding model of evolutionary success in plant species [10,11,13]. Most of the oaks are dominant tree species in forests across the Northern Hemisphere and have significant genetic and ecological values [11,27,33,34]. In this study, we integrated long-read HiFi sequencing data and the Hi-C procedure for the de novo chromosome-level genome assembly of a Chinese endemic and subalpine evergreen oak species, *Q. oxyphylla*. With three rounds of strict refinement of the assembly, we generated a final genome assembly with 12 putative chromosomes and a genome size of 824.15 Mb for *Q. oxyphylla* (Figure 1a and Table 1). The estimated scaffolds N50 and N90 of the obtained genome were 69 and 47 Mb (Table 1), respectively, indicating a well-assembled oak genome in terms of quality when compared with some recently presented oak genomes, such as those for *Q. rubra* (scaffold N50 = 56.1 Mb), *Q. variabilis* (scaffold N50 = 64.9 Mb) and *Q. mongolica* (scaffold N50 = 66.7 Mb) [19,20,22]. According to the quality assessments on the genome assembly, including Hi-C interaction results and evaluated values of BUSCO (>98%) (Figure 1c,d), the final assembled genome of *Q. oxyphylla* is of good quality with high levels of genome completeness. Furthermore, based on the LAI value (18.39) of the genome assembly (Table 2), the new oak genome generated in this study can be considered a reference genome for the study of genomic evolution in *Q. oxyphylla* as well as oak relatives from *Quercus* section *Ilex*.

The results of the genome annotation revealed 62.83% repeat sequences included in the *Q. oxyphylla* genome, of which the *Copia* (12.53%) and *Gypsy* (15.35%) subfamilies of LTR retroelements were the most common (Table 3), reflecting the importance of transposable elements (TEs) in driving genomic evolution, genetic differentiation and adaptation of plant species [35,36]. The proportion of total repeat sequences identified in the genome assembly of *Q. oxyphylla* is higher than that found in some published Chinese oak genomes, for example, *Quercus gilva* (60.20%), *Q. mongolica* (53.75%) and *Q. acutissima* (48%), but lower than that in *Q. variabilis* (67.60%) [20,22,24,37]. Apart from different sequencing techniques used in the genome assembly, this scenario plausibly suggests varying insertion patterns of TEs in the genomes of oak species [35]. The genome annotation of *Q. oxyphylla* indicated 53,730 protein-coding genes in the assembled genome, while 93.45% of these genes were approved as functional against five protein databases (Figure 1e and Table S2). The identified numbers of protein-coding genes in the *Q. oxyphylla* genome assembly in this study are much higher than those found in some important oak species, for example, *Q. robur* (25,808) [13], *Q. lobata* (39,373) [17] and *Q. rubra* (33,333) [19], but are close to the predicted gene numbers in recently published genomes of *Q. variabilis* (54,606) [22], *Quercus rex* (57,279) and *Quercus sichourensis* (54,045) [26]. An explanation of this could be the different annotated methods and filtering strategies adopted for the predictions of protein-coding genes among these studies [16,17]. Given that a BUSCO analysis on the annotated genes indicated a high level of completeness (98.14%), the predicted gene set in the *Q. oxyphylla* genome assembly in this study is believed to be reliable. Additionally, we found more intact LTR-RTs in the *Q. oxyphylla* genome than those of three related oak species (Table S4). All of these results suggest that the HiFi long-read sequencing shows a high accuracy of sequence capture and coverage in genome assemblies, and has been frequently applied in genome and pangenome researches for the unveiling of mechanisms in genomic evolution and adaptation of plant species [6,9,38,39,40].

The analyses of WGD and LTR-RT insertion events indicated similar patterns of genomic evolution in *Q. oxyphylla* to three oak representatives including *Q. glauca*, *Q. robur* and *Q. variabilis* (Figure 2). According to the WGD analysis, no species-specific WGD has occurred in *Q. oxyphylla* or in *Q. glauca*, *Q. robur* and *Q. variabilis*, which potentially confirms the conservative evolution of chromosomes in oak genomes during lineage diversification of the genus *Quercus* [13,35]. The time-calibrated phylogeny generated in this study strongly corroborates the robust framework phylogeny of oaks conducted with genomic datasets [11,33,34]. According to the phylogenetic results, the divergence time of *Q. oxyphylla* from *Quercus* section *Ilex* was inferred at about 20.4 Ma (Figure 3b), which locates it in the estimated time ranges of species diversification for Chinese *Ilex* oaks from a previous phylogenetic study [31]. In addition, if the estimated mean time of 24.9 Ma is considered a split between *Q. oxyphylla* and *Q. glauca* in the phylogeny (Figure 3b), then the time of WGD events in *Q. oxyphylla* and the three other oak representatives in this study can be inferred to be about 124.5 Ma (Ks = 0.2, Figure 2a), which strongly corresponds to the time of ancient whole-genome triplication (γ) in core eudicots around 120 Ma [41]. Therefore, the early WGD events that occurred in the ancestor of oaks may have played important roles in triggering species adaptation and lineage diversification of the genus *Quercus*. The analysis of LTR-RT insertion events based on intact *Copia* and *Gypsy* sequences showed the recent explosion and accumulation of TEs in the genome assembly since the late Miocene (<7 Ma) (Figure 2c,d), a time later than the estimated species differentiation of *Q. oxyphylla* in the phylogeny (Figure 3b). It is plausible that the intense uplift of the Himalayas since about 10 Ma as well as the descending temperatures during the late Miocene could have driven the adaptive evolution of evergreen oak species including *Q. oxyphylla* [31,32,42]; as a genetic consequence, TEs have accumulated rapidly in the genome in order to tackle potential adaptive differentiation. In addition, as reticular evolution among closely related oak species is common due to frequently interspecific introgression and incomplete reproductive isolation [10,12,23,43], TEs may be dispersed and fixed rapidly in the genome among closely related oaks by genetic hitchhiking [6,43,44]. However, the latter hypothesis requires detailed tests on the identification of TEs in relation to introgression sites in the genomes between closely related oaks.

Comparative analysis on orthogroups among eight related oak species and the outgroup *F. sylvatica* in the phylogenetic tree indicated 2074 and 903 gene families underlying expansion and contraction in the assembled genome of *Q. oxyphylla* (Figure 3). Among the significantly expanded gene families, we identified notable numbers of R-gene candidates across the *Q. oxyphylla* genome, such as the NB-LRR genes, LRR receptor-like genes and RLKs genes (Table S6). Additionally, some of the significantly expanded gene families in the *Q. oxyphylla* genome were assigned to a plant–pathogen interaction pathway in the KEGG enrichment analysis (Figure 4c and Table S10). The expansion of disease resistance-related gene families is also found in previously published genomes of *Q. robur*, *Q. lobata* and *Q. rubra*, while the maintenance of high numbers of R-genes in the genome is thought to be induced by high pressures in species habitats, such as pests and pathogens [13,18,19]. Moreover, the GO enrichment analysis revealed that some of the identified gene families were notably assigned to responses to specific circumstances in species habitats, for example, functions in biological processes of the expanded gene families associated with response to toxic substance and stimulus and transmembrane transport of obsolete inorganics in environments (Figure 4a and Table S8). It is plausible that the expansion of gene families in relation to harsh environments might reinforce species-specific adaptation of *Q. oxyphylla* in dry limestone habitats in the face of drastic environmental changes and human activities. However, the above inferences on genomic adaptation of *Q. oxyphylla* require further verifications with comparative genomic analysis on more closely related *Ilex* oak species as well as genome–environment associations across populations within species distribution ranges. Nevertheless, given the possibly adaptative lag in long-lived oak species caused by relatively low evolutionary rates [45,46], the conservation of species habitats is prerequisite to the genetic conservation of *Q. oxyphylla*.

In conclusion, we have presented a chromosome-level de novo genome assembly of the Chinese endemic *Q. oxyphylla* using HiFi long-read sequencing and Hi-C approaches in this study. The annotated results on the high-quality genome assembly including repeat sequences and protein-coding genes as well as orthologous gene clusters have provided critical information for our understanding of genomic evolution in *Q. oxyphylla*, such as WGD and LTR insertion events, as well as its species evolutionary history. Candidate gene families with significant expansion and contraction in the *Q. oxyphylla* genome have been identified and assigned to some important biological processes that plausibly associate with species-specific habitat features and genomic adaptation of the oak species. The new genome assembly of *Q. oxyphylla* generated in this study may provide a valuable genome resource for future genetic studies on evergreen subalpine oak species in *Quercus* section *Ilex* at the genomic level, and facilitate genetic conservation and management of this endemic and threatened oak species.

## 4. Materials and Methods

### 4.1. Sample Information

For genomic library sequencing, fresh young leaves of *Q. oxyphylla* were collected from an adult tree located in Baishi Valley at Lveyang County of Shaanxi Province (33°24′ N, 106°11′ E) in September 2024. A mixture of tissues including adult and young leaves and young stems were sampled from the same tree for transcriptome sequencing. All samples were preserved in liquid nitrogen after collection and stored at −80 °C in a laboratory for genomic DNA and RNA extraction.

### 4.2. Genomic Data Sequencing

High-quality genomic DNA was extracted and prepared for the PacBio long-read HiFi sequencing following standard protocols at Biomarker Technologies Co., Ltd. (Beijing, China). Genome libraries were sequenced using a CCS method in two cells on the PacBio Sequel II platform. The quality of the generated CCS reads was then evaluated and filtered for the genome assembly of *Q. oxyphylla*.

The Hi-C library was implemented by Biomarker Technologies Co., Ltd. (Beijing, China) with protocols described as follows: leaf tissues of *Q. oxyphylla* were fixed with 2% formaldehyde for 20 min to cross-link genomic DNA in cells, and then, genomic DNA was extracted and digested into fragments with HindIII. The ends of the DNA fragments were marked with biotin and then ligated, and the biotinylated DNA fragments were purified and sheared to a fragment size of 300 to 700 bp for subsequent sequencing. The quality of the paired-end Hi-C library was evaluated using the Qubit 2.0 Fluorometer system (Thermo Fisher Scientific Inc., Waltham, MA, USA) and then sequenced on a DNBSEQ platform (MGI Tech, Shenzhen, China) to generate clean raw reads for genome assembly.

Total RNA was extracted for each tissue (adult and young leaves and young stems) with an RNA Extraction Kit (TIANGEN, Beijing, China) following the operation manual. The quality of each RNA sample was assessed on the Qubit 2.0 Fluorometer system (Thermo Fisher Scientific Inc., Waltham, MA, USA). After quality evaluation, a paired-end 150 bp RNA sequencing library was conducted with mixed RNA samples using the TruSeq Stranded mRNA Library Prep Kit for Illumina and then sequenced on the NovaSeq 6000 platform (Illumina Inc., San Diego, CA, USA). The obtained raw reads were checked and used for de novo transcriptome assembly and genome annotation of *Q. oxyphylla*.

### 4.3. Genome Survey, De Novo Assembly and Quality Assessment

The genome size of *Q. oxyphylla* was initially estimated using GenomeScope v2.0 [47] with K-mer frequencies based on the long-read HiFi sequencing dataset. The de novo genome assembly was performed on the CCS long reads using Hifiasm v0.15.1 [48] to generate a primary assembly for the studied oak species. We then integrated the purge_dups pipeline [49] and minimap2 v2.26 program [50] to correct and purge redundant contigs in the primary genome based on read coverage and mapping rates, and a polished draft genome was obtained for subsequent chromosome anchoring. Finally, the clean Hi-C reads were mapped to the draft assembly in juicer v1.6 [51] in order to generate a merged genome file, and then modified using the 3D-DNA pipeline [52] with manual refinements for contigs and scaffolds anchoring on putative chromosomes of the final genome assembly.

The de novo transcriptome assembly was implemented with raw reads from the RNA sequencing library in Trinity v2.14.0 [53]. The obtained unigenes were then filtered with CD-HIT v4.8.1 [54] to remove redundancy and generate unique transcripts for genome annotation.

The quality of the chromosome-level genome assembly was assessed using a heatmap of modified Hi-C results with the plotHicGenome tool (https://github.com/Atvar2/plotHicGenome) (accessed on 11 March 2025). The pattens of interactions among chromosomes in the final genome assembly were evaluated according to the Hi-C heatmap. We also revealed the level of genome completeness on the final assembly with BUSCO v5 [55] and LAI [56] estimations after genome annotation of *Q. oxyphylla*.

### 4.4. Genome Annotation

For the annotation of repetitive sequences in the genome assembly, we adopted RepeatModeler v2.0.2 [57] to identify repeats de novo in the genome; the output data of RepeatModeler v2.0.2 was applied as a repeats database to screen for repetitive sequences in the assembled genome of *Q. oxyphylla* with the program RepeatMasker v4.1.2 [58].

Identifications of protein-coding genes in the genome assembly were performed with three strategies including ab initio, homology-based and transcriptome-based gene predictions. For the ab initio approach, GeneMark-ETP v1.02 [59] and AUGUSTUS v3.5.0 [60] were integrated to create accurate training models on gene predictions with the transcriptome assembly of *Q. oxyphylla*; the Braker3 pipeline [61] was then used to combine gene sets predicted by the training models from the repeat-masked genome assembly. For the homology-based gene prediction, protein sequences of *Arabidopsis thaliana* (L.) Heynh., *Q. robur* and *Q. suber* were obtained and aligned to the assembled genome with miniprot [62] to identify protein-coding genes. The transcriptome-based gene prediction was performed with PASA v2.5.3 [63], which mapped the transcripts of RNA sequencing to the repeat-masked genome assembly for the annotation of protein-coding genes. Final annotations of protein-coding genes predicted from the above strategies were integrated using EVidenceModeler (EVM) v2.1.0 [64].

Functional gene annotation was conducted on protein sequences extracted from the assembled genome of *Q. oxyphylla*. The protein sequences were aligned against five publicly available databases including GO [65], KEGG (Release 115.0) [66], NR (www.ncbi.nlm.nih.gov) (accessed on 14 May 2025), SwissProt [67] and TrEMBL [68] to seek the best hits for each gene and assess gene functions.

For the identification of non-coding RNAs in the genome assembly, we used tRNAscan-SE v2.0 [69] and Barrnap software (https://github.com/tseemann/barrnap) (accessed on 18 May 2025) with eukaryotes as a reference to search and annotate tRNAs and rRNAs, respectively.

### 4.5. Analyses of WGD and LTR-RT Insertion Events

The wgd v1.1 pipeline [70] was adopted to identify homologous gene pairs in the genome within species as well as between species pairs, and calculate values of Ks for the determination of possible WGD in *Q. oxyphylla*. The genome assembled in this study was compared with three oak genomes from *Q. robur*, *Q. variabilis* and *Q. glauca* to infer potential WGD events from the distributions of Ks; the genome of grapevine was also compared because the grape species has only experienced an ancient whole-genome triplication event without any additional whole-genome duplication among core eudicots [41].

The intact LTR-RTs including *Copia* and *Gypsy* elements of the five genomes used in the WGD analysis were initially detected using the LTRharvest [71] and LTR_Finder [72] tools; the identified LTR-RT results were then analyzed with the LTR_retriever v2.9.0 program [73] to estimate the LAI value for quality assessment of the genome assembly as well as to infer potential insertion events of LTR-RTs based on the mutation rates of nucleotides in genome. We considered the mutation rates of 2.5 × 10^−9^ and 9.65 × 10^−9^ for the timing of LTR-RT insertions in grape [74] and oak species [75], respectively.

### 4.6. Ortholog Identification and Phylogenetic Analysis

Protein sequence data of *F. sylvatica* and seven related oak species (*Q. dentata*, *Q. lobata*, *Q. mongolica*, *Q. suber*, *Q. variabilis*, *Q. glauca* and *Q. robur*) representing the major infrageneric sections of the genus *Quercus* were obtained and then jointed with annotated protein-coding sequences from the *Q. oxyphylla* genome to identify orthologous gene clusters using an all-vs-all comparison strategy in the OrthoFinder v2.5.5 program [76].

Phylogenetic analysis was performed on the nine selected species using concatenated single-copy genes identified in the orthologous gene clusters. We constructed a maximum likelihood tree with 1000 bootstrap replicates in the RAxML v8.2.12 software [77] based on a JTT + I + G4 + F model selected by ModelTest-NG v0.1.7 [78]. Divergent times on the phylogeny were estimated using the MCMCtree module in the PAML v4.10.7 software package [79] with a time calibration around 25.2 to 77.4 Ma on the root divergence between *F. sylvatica* and oaks, which were obtained from the TimeTree web database (www.timetree.org) (accessed on 2 June 2025).

Based on the time-calibrated phylogenetic tree, the CAFE5 software [80] was further adopted with a GAMMA model to evaluate potential expansion and contraction of the shared orthologous gene families among the nine studied species. Finally, functional enrichment analyses on significantly expanded and contracted gene families detected in the genome assembly of *Q. oxyphylla* were performed against the GO and KEGG database in the TBtools-II program [81] to explore potential adaptive variation during species evolution of *Q. oxyphylla*.

## Figures and Tables

**Table 1:** Summary of assembled results on the genome of *Q. oxyphylla*.

Characteristic	Preliminary Assembly	Non-Redundant Assembly	Chromosome-Level Assembly
Contig/Scaffold N50 (bp)	58,005,987	57,934,126	69,436,167
Contig/Scaffold N90 (bp)	10,092,746	33,605,831	47,624,653
Largest Contig/Scaffold (bp)	102,844,174	102,844,174	102,838,673
GC Content (%)	36.61	36.27	35.74
Genome Size (bp)	896,487,760	855,227,807	824,148,311

**Table 2:** Summary statistics of genome assembly on each chromosome for *Q. oxyphylla*.

Chromosome	Length (bp)	GC Content	Gene Number	LAI
Chr1	96,627,000	35.46%	5619	17.16
Chr2	80,058,500	35.60%	4962	13.87
Chr3	91,819,500	35.65%	5829	21.96
Chr4	102,838,673	35.92%	7076	19.85
Chr5	62,517,000	35.59%	4199	14.12
Chr6	47,624,653	35.57%	3352	23.50
Chr7	40,678,847	35.84%	2937	16.82
Chr8	69,136,267	35.79%	4639	23.52
Chr9	56,816,233	36.37%	3547	23.75
Chr10	57,994,500	35.59%	3819	18.87
Chr11	55,916,000	35.87%	3891	22.09
Chr12	62,121,138	35.61%	3860	21.98
Overall	824,148,311	35.74%	53,730	18.39

LAI: Long terminal repeats (LTR) assembly index.

**Table 3:** Summary statistics of annotated repeat sequences in genome assembly of *Q. oxyphylla*.

Type	Number	Length (bp)	Percentage (%)
Retroelements	377,407	306,686,132	37.21
SINEs	7085	1,824,306	0.22
Penelope	875	230,372	0.03
LINEs	68,429	36,773,313	4.46
L2/CR1/REX	3852	1,108,871	0.13
RTE/Bov-B	14,685	3,217,655	0.39
L1/CIN4	47,300	31,852,452	3.86
LTR	301,893	268,088,513	32.53
BEL/Pao	428	146,294	0.02
Ty1/*Copia*	104,545	103,303,150	12.53
*Gypsy*/DIRS1	118,002	126,500,466	15.35
Retroviral	3554	1,269,090	0.15
DNA transposons	177,890	47,669,451	5.78
hobo-Activator	63,050	15,662,120	1.90
Tc1-IS630-Pogo	3672	709,778	0.09
Tourist/Harbinger	13,921	4,709,305	0.57
Other (Mirage, P-element, Transib)	3392	850,503	0.10
Rolling-circles	11,423	4,509,545	0.55
Small RNA	4097	1,126,860	0.14
Satellites	2097	1,405,276	0.17
Simple repeats	388,858	13,676,675	1.66
Low complexity	50,223	2,437,895	0.30
Total	1,766,678	514,096,453	62.38

**Table 4:** Results of prediction of protein-coding genes in the genome assembly of *Q. oxyphylla*.

	Ab Initio		Homology			RNA	EVM
	AUGUSTUS	GeneMark	*Arabidopsis thaliana*	*Quercus suber*	*Quercus robur*		
Number of genes	54,291	36,326	48,871	56,849	72,158	17,897	53,730
Average gene length (bp)	3265.62	3204.82	8447.57	5725.26	7425.96	7395.15	3548.05
Average CDS length (bp)	1070.71	1501.83	2689.17	2676.47	3118.51	943.34	1026.24
Average exon length (bp)	248.44	237.97	225.83	323.83	340.84	308.24	257.83
Average intron length (bp)	786.48	956.33	1187.33	1053.14	1217.88	1638	846.17
Average number of exons per gene	4.31	9.34	11.91	8.27	9.15	4.50	3.98

## Data Availability

All data analyses were performed using published bioinformatics software with default settings. The raw data and genome assembly in this work are temporarily private for further data assessment, but can be accessed from the corresponding author.

## Supplementary Materials

The following supporting information can be downloaded at https://www.mdpi.com/article/10.3390/plants15081238/s1: Figure S1: Result of genome survey on *Q. oxyphylla* using a K-mer analysis (K = 21); Table S1: Summary statistics of genomic sequencing libraries conducted for genome assembly of *Q. oxyphylla*; Table S2: Information of functional gene annotation in five protein databases for genome assembly of *Q. oxyphylla*; Table S3: Information on identified homologous gene pairs among five species for whole-genome duplication analysis; Table S4: Genome-wide identification of intact long terminal repeat retrotransposons (LTR-RTs) and two superfamilies (*Copia* and *Gypsy*) in five species for analysis of LTR-RT insertion events; Table S5: Identification results of orthologous gene clusters among nine species in this study; Table S6: Information on candidate genes in the significantly expanded gene families in *Q. oxyphylla* genome; Table S7: Information on candidate genes in the significantly contracted gene families in *Q. oxyphylla* genome; Table S8: GO (Gene Ontology) enrichment analysis of the significantly expanded gene families in *Q. oxyphylla* genome; Table S9: GO (Gene Ontology) enrichment analysis of the significantly contracted gene families in *Q. oxyphylla* genome; Table S10: KEGG (Kyoto Encyclopedia of Genes and Genomes) pathway analysis of the significantly expanded gene families in *Q. oxyphylla* genome; Table S11: KEGG (Kyoto Encyclopedia of Genes and Genomes) pathway analysis of the significantly contracted gene families in *Q. oxyphylla* genome.
