# Supplementary figures and images for: Chromosome-Level Genome Assembly and Comparative Genomic Analysis of Quercus oxyphylla, an Evergreen Subalpine Oak Species Endemic to China
Source: Plants (Basel). 2026 Apr 17;15(8):1238. doi: 10.3390/plants15081238 (PMC13119989; doi:10.3390/plants15081238)

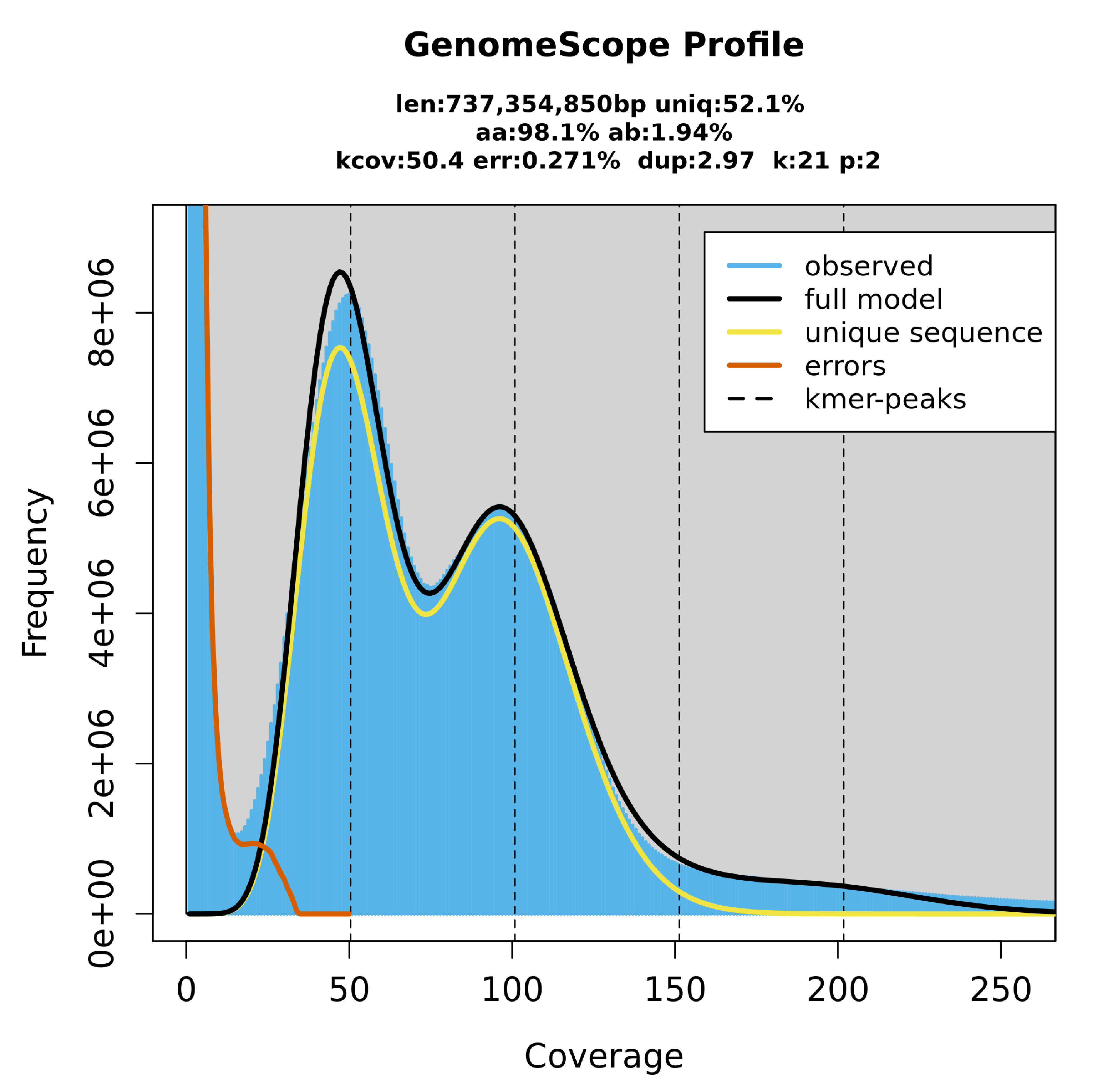

Supplement: Supplementary file 1 [file plants-15-01238-s001.zip › Figure_S1.jpg]
